# Treatment of the Carbuncular Anthrax, with Report of Cases*Read before the Atlanta Society of Medicine, March, 1891.

**Published:** 1891-05

**Authors:** Walter A. Crow

**Affiliations:** Atlanta, Ga.


### TREATMENT OF THE CARBUNCULAR ANTHRAX, WITH REPORT OF CASES.*

BY WALTER A. CROW, M. D., ATLANTA, GA.

*Mr. President and Gentlemen:*

My paper to-night is intended to give the history and treatment of five cases of Carbuncular Anthrax that have come under my care in the last eighteen months.

CASE 1, male adult, laborer, age 23, strong and vigorous, he had an indurated swelling on the back of neck, which had been troubling him for the past three or four days, and at the time of his visit to my office, was suffering intense pain of a sharp and lancinating character. It was evidently a carbuncle. I injected into the center of this mass, which was already beginning to have a bluish tint, 15 or 20 minims of a 20 per cent. carbolized solution with glycerine; also painted over the reddened surface, which was fully three inches in diameter, a sol. of carbolic acid and glycerine aa, and dressed it with lint saturated in 5 per cent. carbolized oil, with directions to apply the dressing anew every morning. I did not see him for more than a week, when he told me that the application gave him nearly entire relief, and he was then almost well.

CASES 2 and 3, have very much the same history, excepting in case No. 3, the patient was having rigors with slight fever. I applied the same treatment of carbolized injections into the mass and a carbolized dressing, also gave tr. ferri chlor., gtt. x, with sulph. quinine 3 gr. ter in die. I should state that this case was more advanced than the two preceding ones; having a duration of nearly a week, and was beginning to have the characteristic little openings around the center that was discharging a thin purplish fluid.

In this case, resolution did not take place as in the other two, but it did relieve the acute pain in a great measure and evidently arrested its further spreading; this patient made a good recovery in at least two weeks after my first seeing him.

CASE 4, Was a married lady, age 55, mother of two children, and was thin and delicate. I was called to see her the first

*Read before the Atlanta Society of Medicine, March, 1891.

time, the 5th of last November. She was suffering intense pain and had considerable fever. I found over the lower end of left scalpular a large indurated swelling with several small openings at the summit that was discharging a sero-pus like fluid. This surface was of a dark purplish color which was followed up by a deep scarlet that gradually faded away, making an enormous surface of inflamed tissue measuring at least twelve inches in diameter. This was by far the worst case of carbuncle that I had ever seen, and on a subject on account of age and general debility, made it very unpromising, on account of the drain I knew must necessarily be from such a mass of inflamed and rotten tissue. It was on the tenth day of its course that I saw her, she was having chills with high fever—103-4 degrees F. I slightly enlarged the opening at the center with my bistory, on account of the rotten appearance of the mass beneath, for they had vigorously applied poultices from the beginning, so I was told. I then took a wooden toothpick and wrapped cotton on it, this I saturated in pure carbolic acid and thoroughly cauterized the opening I had enlarged down to the base of the tumor, and then painted the surface with carbolic acid and glycerine aa, and dressed the entire inflamed surface with lint saturated in Darby's fluid diluted half. I gave a hypodermic of morphae, also ordered a saline to open the bowels and prescribed tr. ferri chlor., gtt. x, sulph. quinine, gr. vi, to be repeated three times a day; also advised milk punch and egg-nog to sustain strength. This dressing I repeated once a day up to November 11th, when on account of the alarming symptoms of blood poison, pyaemic, which were pain in the joints, together with the continued rigors and high fever—105 degrees—also the general giving away of the vital powers.

I decided to make a clean dissection of the entire infected mass and give her the benefit of the chance, which I did in two operations, because my patient I thought was too weak to take ether, so I gave her about an ounce of whisky and injected sulph. morphae, gr. ss, sulph. atropine, 1-100, and then applied a 4 per cent. of cocaine, injected in the mass I expected to remove; at the first operation I removed about half the mass, which was finished up the following day, 24 hours afterwards. This was done under as strict antiseptic precautions as I could carry out, using bichloride sol., 1-1000. The entire incision when completed on the second day measured five inches long and three across ; this was thoroughly washed out with the bichlor., then dusted with iodoform and packed with lint that had been saturated with the bichlor. sol. and squeezed out. On the morning following the last operation the temperature dropped down to 101 degrees F., and no return of the rigors ; the second day following, the temperature was below 100 degrees, and my patient was feeling "pretty well," as she expressed it. I should also state in this connection that there was a collection of pus under the left axilla about four inches from my incision ; at the time I operated this, I tried to aspirate, but failed on account of the pus being so thick, so I made an incision and evacuated about 1 1-2 ounces of thick laudable pus, then washed out the cavity well with the bichlor. sol.; this healed promptly after two or three washings. After I had removed all of the diseased tissue, I found that the adipose tissue adjacent was more or less infiltrated with pledgets of pus ; this I could remove by gently pressing on the surface in the direction of the incision, and on account of this infiltration of pus, I left the wound open for three or four days until I could remove all of this pus. I then brought the edges as near together as I possibly could with a continued suture of carbolic silk, and allowed the wound to heal by granulation. On the 5th day—November 16th, the redness had entirely left the surface up to the margins of the wound, which was beginning to take on healthy granulations. My patient's condition continued to improve ; she suffered no pain after the operation, only the soreness necessarily on movement and in dressing.

The antiseptic dressing was repeated every day for the first week, then at longer intervals as there was a very small discharge of pus. She was discharged December 27th, the wound had entirely healed up and her general health was fully as good, or better than it had been for the past year or so.

CASE 5, was a physician of this city ; he had suffered from a very large carbuncle on the back of his neck more than a year ago. It had been treated with carbolized injections, but not followed with the carbolized dressing. This case came under my observation about January 1st, 1890. There was considerable induration with redness of the surface, together with ten or twelve little boils or feruncles scattered over the swollen mass. In this case I advised cauterizing these little boils by applying pure carbolic acid on a wooden tooth-pick, passing it through the little openings well down to the base; this was done, after which the carbolized dressing was employed and was followed by almost entire relief of the throbbing pain in three or four hours. I also advised him to treat any other pimples that might come in the same way; one application was always sufficient, and in a week's time the swelling had all subsided and he was again appaıently well.

REMARKS.—While I do not propose to consider the germ factor of this disease in an etiological sense, but merely to report these cases and give the results of my observations, not that there will be anything new from what is already in vogue with the profession, but merely to add my testimony to what I consider the most rational treatment for these painful and often serious troubles.

1st. I believe that the majority of cases from one to three day's duration can be aborted by the carbolic acid treatment as above stated, and

2d. That in those cases that have advanced so far that more or less destruction of tissue has taken place, still we may do a great deal of good by this same treatment in relieving pain and cutting short the usual course if left to itself, or under any other treatment, except it be

3d. By dissecting out and removing the entire diseased mass. This last measure I would strongly advise in all cases in weak and feeble persons where the disease had advanced to the purplish discoloration, which indicates to a certain extent the destruction of the underlying tissue. Suffice it to say I, in connection with this removal of diseased tissue, advise the strictest antiseptic measures, not only as a means of healing according to the most approved surgery of the day, but for destroying the disease germ, and thereby preventing the tendency after of a return of the trouble, or as a precaution against the secondary eruption of boils which are so common in this trouble if left to run its usual course.

